# Derivatives of 9-phosphorylated acridine as butyrylcholinesterase inhibitors with antioxidant activity and the ability to inhibit β-amyloid self-aggregation: potential therapeutic agents for Alzheimer’s disease

**DOI:** 10.3389/fphar.2023.1219980

**Published:** 2023-08-09

**Authors:** Galina F. Makhaeva, Nadezhda V. Kovaleva, Elena V. Rudakova, Natalia P. Boltneva, Sofya V. Lushchekina, Tatiana Yu Astakhova, Elena N. Timokhina, Olga G. Serebryakova, Alexander V. Shchepochkin, Maxim A. Averkov, Irina A. Utepova, Nadezhda S. Demina, Eugene V. Radchenko, Vladimir A. Palyulin, Vladimir P. Fisenko, Sergey O. Bachurin, Oleg N. Chupakhin, Valery N. Charushin, Rudy J. Richardson

**Affiliations:** ^1^ Institute of Physiologically Active Compounds at Federal Research Center of Problems of Chemical Physics and Medicinal Chemistry, Russian Academy of Sciences, Chernogolovka, Russia; ^2^ Emanuel Institute of Biochemical Physics, Russian Academy of Sciences, Moscow, Russia; ^3^ Institute of Organic Synthesis, Russian Academy of Sciences, Yekaterinburg, Russia; ^4^ Department of Organic and Biomolecular Chemistry, Ural Federal University, Yekaterinburg, Russia; ^5^ Department of Chemistry, Lomonosov Moscow State University, Moscow, Russia; ^6^ Department of Pharmacology of the Institute of Biodesign and Complex System Modeling of Biomedical Science & Technology Park of Sechenov I.M., First Moscow State Medical University, Moscow, Russia; ^7^ Department of Environmental Health Sciences, University of Michigan, Ann Arbor, MI, United States; ^8^ Department of Neurology, University of Michigan, Ann Arbor, MI, United States; ^9^ Center of Computational Medicine and Bioinformatics, University of Michigan, Ann Arbor, MI, United States; ^10^ Michigan Institute for Computational Discovery and Engineering, University of Michigan, Ann Arbor, MI, United States

**Keywords:** 9-phosphoryl-9,10-dihydroacridines, 9-phosphorylacridines, acetylcholinesterase, butyrylcholinesterase, Alzheimer’s disease

## Abstract

We investigated the inhibitory activities of novel 9-phosphoryl-9,10-dihydroacridines and 9-phosphorylacridines against acetylcholinesterase (AChE), butyrylcholinesterase (BChE), and carboxylesterase (CES). We also studied the abilities of the new compounds to interfere with the self-aggregation of β-amyloid (Aβ_42_) in the thioflavin test as well as their antioxidant activities in the ABTS and FRAP assays. We used molecular docking, molecular dynamics simulations, and quantum-chemical calculations to explain experimental results. All new compounds weakly inhibited AChE and off-target CES. Dihydroacridines with aryl substituents in the phosphoryl moiety inhibited BChE; the most active were the dibenzyloxy derivative **1d** and its diphenethyl bioisostere **1e** (IC_50_ = 2.90 ± 0.23 µM and 3.22 ± 0.25 µM, respectively). Only one acridine, **2d**, an analog of dihydroacridine, **1d**, was an effective BChE inhibitor (IC_50_ = 6.90 ± 0.55 μM), consistent with docking results. Dihydroacridines inhibited Aβ_42_ self-aggregation; **1d** and **1e** were the most active (58.9% ± 4.7% and 46.9% ± 4.2%, respectively). All dihydroacridines **1** demonstrated high ABTS^•+^-scavenging and iron-reducing activities comparable to Trolox, but acridines **2** were almost inactive. Observed features were well explained by quantum-chemical calculations. ADMET parameters calculated for all compounds predicted favorable intestinal absorption, good blood–brain barrier permeability, and low cardiac toxicity. Overall, the best results were obtained for two dihydroacridine derivatives **1d** and **1e** with dibenzyloxy and diphenethyl substituents in the phosphoryl moiety. These compounds displayed high inhibition of BChE activity and Aβ_42_ self-aggregation, high antioxidant activity, and favorable predicted ADMET profiles. Therefore, we consider **1d** and **1e** as lead compounds for further in-depth studies as potential anti-AD preparations.

## 1 Introduction

This paper represents a continuation of our ongoing program of drug discovery for neurodegenerative conditions, with a particular emphasis on Alzheimer’s disease (AD). We have focused on AD for several reasons. For example, among the various types of age-related dementias, AD is the most prevalent. Moreover, AD is devastating for patients and their caregivers. It begins with memory loss, progresses to steady declines in cognitive function, and ultimately results in total disability and death (Calabro et al., 2021). Furthermore, there is no cure; treatment has been limited to partial alleviation of symptoms using the few drugs thus far approved by the FDA (Huang and Mucke, 2012; Cummings et al., 2019).

A major impediment to progress in drug discovery for AD is that the disease does not have a single cause; rather, it is multifactorial (Carreiras et al., 2013; Bachurin et al., 2017). Accordingly, our approach is to combine into single molecules the capabilities to inhibit several of the biological macromolecules or processes involved in the pathogenesis of the disease (Makhaeva et al., 2019c; Bachurin et al., 2023). In the present investigation, we have included the following targets: cholinergic neurotransmission, oxidative stress, and Aβ aggregation, as discussed in turn below.

One of the hallmarks of AD pathology is the demise of cholinergic neurons (Ballard et al., 2005; Hampel et al., 2019). Consequently, during the clinical course of the disease, the activity of acetylcholinesterase (AChE) decreases. In an apparent attempt to compensate for the loss of AChE activity, the activity of butyrylcholinesterase (BChE) increases (Ballard et al., 2005). Nevertheless, in the AD brain, there is an overall deficit of the neurotransmitter, acetylcholine (ACh). Therefore, inhibitors of one or both cholinesterases have been used to increase ACh concentrations in an effort to offset cognitive decline due to impaired cholinergic neurotransmission (Lane et al., 2006; Macdonald et al., 2014). Unfortunately, inhibition of cholinesterase activity on its own is insufficient to halt or retard the underlying neuropathic processes (Martinez and Castro, 2006; Agatonovic-Kustrin et al., 2018).

Among the mechanisms thought to contribute to AD pathogenesis is oxidative stress, which can lead to neuronal death by damaging the lipid and protein constituents of mitochondria, other organelles, and plasma membranes. This situation arises when the concentrations of reactive oxygen species (ROS) in susceptible sites reach critical levels due to their rates of production exceeding their rates of removal (Ortiz et al., 2017; Pohanka, 2018). Although antioxidant processes in the brain decrease with increasing age, these processes are further compromised in AD brain. Consequently, antioxidants are considered to be of adjunctive value in treating AD (Moreira et al., 2005). Indeed, antioxidant properties can be incorporated into drug candidates that contain pharmacophores directed against other targets, such as cholinesterases (Rosini et al., 2011; Ismaili and Romero, 2017; Makhaeva et al., 2020).

Accumulation of Aβ plaques is another process that has been amply linked to AD pathology (Walsh et al., 2005; Castellani et al., 2019; Jeremic et al., 2021). In view of this, under its new accelerated approval program, the FDA has recently approved two monoclonal antibody drugs that could potentially attenuate AD progression by clearing Aβ plaques from the brain (Vaz et al., 2022; van Dyck et al., 2023). However, these approvals have been controversial owing to concerns regarding safety and efficacy (Reardon, 2023). Another approach is to understand the mechanisms of formation of neurotoxic Aβ oligomers and to design compounds to intervene (Jeremic et al., 2021), which was the strategy we employed in our present investigation.

For the current work, we chose acridines as our core structures for building potential multifunctional agents. Our rationale included the fact that in drug discovery for neurodegenerative diseases, acridines are considered to be privileged scaffolds (Bongarzone and Bolognesi, 2011). Moreover, derivatives of acridine have proved to exert therapeutic effectiveness against such diverse illnesses as bacterial infections (Wainwright, 2001), malaria (Girault et al., 2000), trypanosomiasis and leishmaniasis (Gamage et al., 1997), viral infections (Lyakhov et al., 2000), cancer (Denny, 2002), inflammation and diabetes (Sondhi et al., 2010; Mallu et al., 2017) and prion diseases (Korth et al., 2001; Klingenstein et al., 2006; Collinge et al., 2009). They also serve as ideal points of departure for the generation of novel multitarget lead compounds and drug candidates for neurodegenerative and other conditions (Csuk et al., 2009; Antosova et al., 2011; Bongarzone and Bolognesi, 2011). Furthermore, it is well established that numerous compounds containing an acridine moiety display potent inhibitory activity toward AChE and BChE (Kozurkova et al., 2011; Jin, 2014). Tacrine, (9-amino-1,2,3,4-tetrahydroacridine), an especially well-known acridine-based cholinesterase inhibitor, was the first FDA-approved drug for the treatment of the cognitive symptoms of AD (Crismon, 1994). Along with tacrine, the acridine template has been employed for the design and development of new multifunctional molecules as potential therapeutic agents for AD (Makhaeva et al., 2017; Sharma and Piplani, 2017; Chufarova et al., 2018; Lotfi et al., 2020; Tseng et al., 2020; Maciejewska et al., 2022).

In particular, in our previous work, we studied the esterase profile and estimated the antioxidant activity (AOA) of four groups of new 9-substituted acridine derivatives (Makhaeva et al., 2017). We demonstrated that 9-amino-*N*-methyl-9,10-dihydroacridine derivatives combined effective inhibition of AChE and BChE with a high level of radical-scavenging activity.

The aim of the present investigation was to study the possibility of creating multifunctional agents for AD therapy based on new 9-phosphoryl-9,10-dihydroacridines and 9-phosphorylacridines with various structures of the phosphoryl fragment, obtained using our original strategy of functionalization of acridines by nucleophilic aromatic substitution of hydrogen (S_N_^H^) (Chupakhin et al., 1994; Charushin and Chupakhin, 2004; Chupakhin and Charushin, 2017). We were encouraged to pursue 9-phosphoryl derivatives by the observation that connecting acridine with a phosphorus moiety could enhance penetration of acridines through cell membranes (Demkowicz et al., 2016). In addition, recently studied *N*- and *O*-phosphorylated tacrines (Przybylowska et al., 2022) demonstrated high anti-AChE and anti-BChE activities, and some of them had reduced hepatotoxicity compared to tacrine.

In summary, we chose to investigate the effects of our new compounds on the components of AD pathogenesis outlined above: cholinergic neurotransmission, oxidative stress, and Aβ aggregation. Accordingly, we determined the following characteristics of the synthesized compounds: the esterase profile, i.e., the inhibitory activities against AChE, BChE, and a structurally related off-target carboxylesterase (CES, EC 3.1.1.1); inhibition of Aβ self-aggregation; and antioxidant activities in the ABTS and FRAP assays. We then analyzed the observed effects using quantum chemical calculations and computational molecular modeling. Finally, we carried out computational predictions of ADMET properties.

## 2 Materials and methods

### 2.1 Synthesis of compounds

**FIGURE 1:**
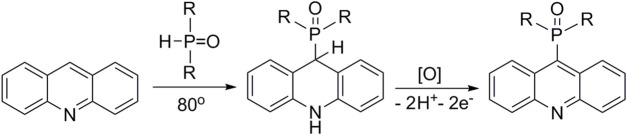
Synthesis of 9-phosphoryl-9,10-dihydroacridines and 9-phosphorylacridines.

9-Phosphoryl-9,10-dihydroacridines **1a–f** and 9-phosphorylacridines **2a–f** were obtained according to Figure 1 as described in our recent publication (Shchepochkin et al., 2021).

### 2.2 Biological investigations

Experiments were performed in alignment with the standard operating procedures approved by IPAС RAS as described below.

#### 2.2.1 *In vitro* inhibition of AChE, BChE, and CES activities

Enzymes, substrates, and reference compounds were from Sigma-Aldrich (St. Louis, MO, United States). The activity of enzymes was measured spectrophotometrically, as described in detail in (Makhaeva et al., 2017) using ATCh iodide, BTCh iodide, and 4-NPA as substrates for AChE, BChE, and CES, respectively. Experimental conditions: K,Na-phosphate buffer (100 mM), 25°С, pH 7.5 for AChE and BChE and pH 8.0 for CES assay. Measurements were carried out on a BioRad Benchmark Plus microplate spectrophotometer (Hercules, CA, United States).

Test compounds were dissolved in DMSO; final concentration of solvent in the incubation mixture was 2% (v/v). Initial assessment of inhibitory activity was carried out by determining the degree of enzyme inhibition at a compound concentration of 20 µM. For the most active compounds (inhibition ≥35%), IC_50_ values were determined.

Mechanism of BChE inhibition was assessed by a detailed analysis of enzyme kinetics with three increasing concentrations of inhibitor and six substrate concentrations as described in detail in Makhaeva et al. (2017).

#### 2.2.2 Inhibition of β-amyloid (1-42) (Aβ_42_) self-aggregation

Inhibition of Aβ_42_ self-aggregation by test compounds was studied using the thioflavin T (ThT) fluorescence method (LeVine, 1999; Bartolini et al., 2003; Munoz-Ruiz et al., 2005) with minor modifications as described in detail in (Makhaeva et al., 2023). Lyophilized HFIP-pretreated Aβ_42_ (AnaSpec Inc., 0.5 mg) was dissolved in DMSO to obtain a stable 500 μM solution. The samples of 50 μM Aβ_42_ in 215 mM Na-phosphate buffer pH 8.0 were incubated for 24 h at 37°C in the absence or presence of 100 µM test compounds. Myricetin and Propidium iodide in the same concentration were used as references. After incubation, 5 μM ThT in 50 mM glycine-NaOH buffer pH 8.5 was added, and the fluorescence was measured at 440 nm (exc.) and 485 nm (emis.) with a FLUOStar Optima microplate reader (LabTech, Ortenberg, Germany). Blanks consisted of 215 mM Na-phosphate buffer, pH 8.0, 20% (v/v) DMSO or test compounds, respectively.

The inhibition (%) of Aβ_42_ self-aggregation by the test compounds was calculated using the following equation:

$$\%\;inhibition=100\;–\;\left({IF}_{i}\;/\;{IF}_{o}\right)\times 100,$$

where IF_i_ and IF_o_ are the fluorescence intensities obtained for Aβ_42_ in the presence or absence of inhibitor, respectively, after subtracting the fluorescence of respective blanks.

#### 2.2.3 Antioxidant activity (AOA)

##### 2.2.3.1 ABTS radical cation scavenging activity assay

Radical scavenging activity of the compounds was evaluated using the ABTS radical cation (2,2ʹ-azinobis-(3-ethylbenzothiazoline-6-sulfonic acid), ABTS^•+^) decolorization assay (Re et al., 1999), with minor modifications described in detail in Makhaeva et al. (2019a). To prepare the solution of ABTS^•+^, equal volumes of aqueous solutions of 7 mM ABTS and 2.45 mM potassium persulfate were incubated for 12–16 h at room temperature in the dark. Radical scavenging capacity of the compounds was determined by mixing 10 μL of compound solution in DMSO with 240 μL of ABTS^•+^ working solution in ethanol (100 μM final concentration). The reduction in ABTS^•+^ absorbance was measured spectrophotometrically at 734 nm using a xMark UV/VIS microplate spectrophotometer (Bio-Rad, Hercules, CA, United States) for 1 h with an interval of 10 min compared to a standard synthetic antioxidant Trolox (6-hydroxy-2,5,7,8-tetramethylchroman-2-carboxylic acid). Ascorbic acid was used as a positive control.

The antioxidant activity of the compounds was reported as Trolox equivalent antioxidant capacity (TEAC values) as the ratio of the slopes of the concentration−response curves, test compound/Trolox. The IC_50_ values for the test compounds were also determined.

##### 2.2.3.2 FRAP assay

The ferric reducing antioxidant power (FRAP) assay proposed by Benzie and Strain (1996); Benzie and Strain (1999) modified to be performed in 96-well microplates as described in detail in Makhaeva et al. (2020) was used. 10 μL (0.5 mM) of the tested or reference compounds were mixed with 240 µL of FRAP reagent (2.5 mL of 10 mM TPTZ (2,4,6-tris(2-pyridyl)-s-triazine) solution in 40 mM HCl, 2.5 mL of 20 mM FeCl_3_ in distilled water and 25 mL of 0.3 M acetate buffer pH 3.6); the absorbance of the mixture was measured spectrophotometrically (λ = 593 nm) with a FLUOStar OPTIMA microplate reader at 600 nm after a 1 h incubation at 37°C against a blank. Trolox was used as a reference compound. The results were expressed as Trolox equivalents (TE)—the ratio of the concentrations of Trolox and the test compound resulting in the same effect on ferric reducing activity.

### 2.3 Statistical analyses

Plots, linear regressions, and IC_50_ values were determined using Origin 6.1 for Windows, OriginLab (Northampton, MA, United States). All tests were performed at least in triplicate in three independent experiments. Results are presented as mean ± SEM calculated using GraphPad Prism version 6.05 for Windows (San Diego CA, United States).

### 2.4 Molecular modeling studies

#### 2.4.1 Molecular docking

The Calculator Plugins of MarvinSketch 21.14.0, ChemAxon (https://www.chemaxon.com, accessed on 27 January 2023) and MolGpKa (Pan et al., 2021) (https://xundrug.cn/molgpka, accessed on 27 January 2023) were used to estimate the p*K*_a_ values of the ligands. Because the p*K*_a_ values of the acridine fragments for all considered compounds were estimated to be below 5, all conjugates were utilized for molecular docking in their neutral form. The ligand compounds were optimized using a DFT quantum chemistry method (B3LYP/6-31G*, GAMESS-US (Schmidt et al., 1993) software). For molecular docking, the optimized structures of the ligands were employed, with partial atomic charges obtained from QM results based on the Löwdin scheme (Löwdin, 1970).

The protein targets used for docking included X-ray structures of human AChE co-crystallized with donepezil (PDB: 4EY7, chain A) (Cheung et al., 2012), an optimized X-ray structure of human BChE (PDB: 1P0I) (Nicolet et al., 2003; Masson et al., 2013), and all conformers of the Aβ_42_ solution NMR structure (PDB ID 1IYT) (Crescenzi et al., 2002).

AutoDock 4.2.6 software (Morris et al., 2009) was utilized to perform molecular docking. The docking grid box was set to cover the entire active site gorge of AChE (22.5 Å × 22.5 Å × 22.5 Å) and BChE (15 Å × 20.25 Å × 18 Å), as well as the entire Aβ42 molecule for all conformers (43.5 Å × 28.5 Å × 54.75 Å), with a grid spacing of 0.375 Å used in all cases. The Lamarckian Genetic Algorithm (LGA) (Morris et al., 1998) was used with 256 runs, 25 × 10^6^ evaluations, 27 × 10^4^ generations, and a population size of 3,000. Figures were created using PyMol (www.pymol.org, accessed on 21 July 2016).

#### 2.4.2 Molecular dynamics simulations

For molecular dynamics (MD) simulations, an initial complex of BChE with the most active compound **1d** was taken from molecular docking results. Crystallographic waters found in the initial protein X-ray structure were added, except for ones overlapping with the inhibitor molecule. TIP3P water molecules were added, forming a box with boundaries extending 10 Å beyond the protein using the VMD *solvate* module. Na^+^ and Cl^−^ ions were added up to 150 mM ion concentration, and the neutral state of the whole system was achieved using the VMD *autoionize* module. Force field parameters of the ligand were derived with the CharmGenFF program v. 2.5 (Vanommeslaeghe et al., 2010).

MD simulation was performed with NAMD 3.0b2 software (Phillips et al., 2020) using the CHARMM36 force field (Vanommeslaeghe et al., 2010). During MD simulation, the system was maintained at constant temperature (298 K) and pressure (1 atm, NPT ensemble), using Langevin dynamics and the Nosé-Hoover barostat; the timestep was 1 fs. Periodic boundary conditions and PME electrostatics were applied. Before the productive MD run, a system minimization was conducted during 3,000 steps; next, a 1 ns solvent equilibration run was carried out with the protein and ligand coordinates fixed, and then the structure was fully minimized during 3,000 steps. After that, a 50 ns production run was performed. Analysis of the obtained MD trajectory was done with the VMD 1.9.4a53 package (Humphrey et al., 1996). Molecular modeling figures were prepared with PyMOL.

#### 2.4.3 Quantum-chemical analysis of AOA

Quantum-chemical calculations were performed with the Gaussian 16 (Frisch et al., 2016) and Priroda 19 (Laikov and Ustynyuk, 2005; Laikov, 2020) packages. At the initial stage, the conformational analysis was done in the PBE0 functional (Adamo and Barone, 1999) and TZVP basis set (Schäfer et al., 1994) in the gas phase by the Priroda software. The obtained conformations were optimized using the long-range-corrected functional CAM-B3LYP (Yanai et al., 2004) in the 6-31++G (d,p) basis set (Rassolov et al., 2001) by the Gaussian program. The energies were refined by single-point calculations in the aug-cc-pVTZ basis set (Papajak et al., 2011). Solvent effects were taken into account by applying the Solvation Model based on density (SMD) (Marenich et al., 2009). Six compounds were investigated; namely, three dihydroacridines (**1a, 1c, 1e**) and three acridines (**2a, 2c, 2e**).

#### 2.4.4 Prediction of ADMET, physicochemical, and PAINS profiles

Lipophilicity (LogP_ow_) and aqueous solubility (pS) were estimated by the ALogPS 3.0 neural network model implemented in the OCHEM platform (Sushko et al., 2011). Human intestinal absorption (HIA) (Radchenko et al., 2016), blood–brain barrier distribution/permeability (LogBB) (Dyabina et al., 2016; Radchenko et al., 2020), and hERG-mediated cardiac toxicity risk (channel affinity p*K*_*i*_ and inhibitory activity pIC_50_) (Radchenko et al., 2017) were estimated using the integrated online service for the prediction of ADMET properties. This service implements predictive QSAR models based on accurate and representative training sets, fragmental descriptors, and artificial neural networks. The quantitative estimate of drug-likeness (QED) values (Bickerton et al., 2012) were calculated, and the Pan Assay INterference compoundS (PAINS) alerts were checked using RDKit version 2021.09.2 software.

## 3 Results and discussion

### 3.1 Chemistry. Synthesis of 9-substituted acridines

**FIGURE 2:**
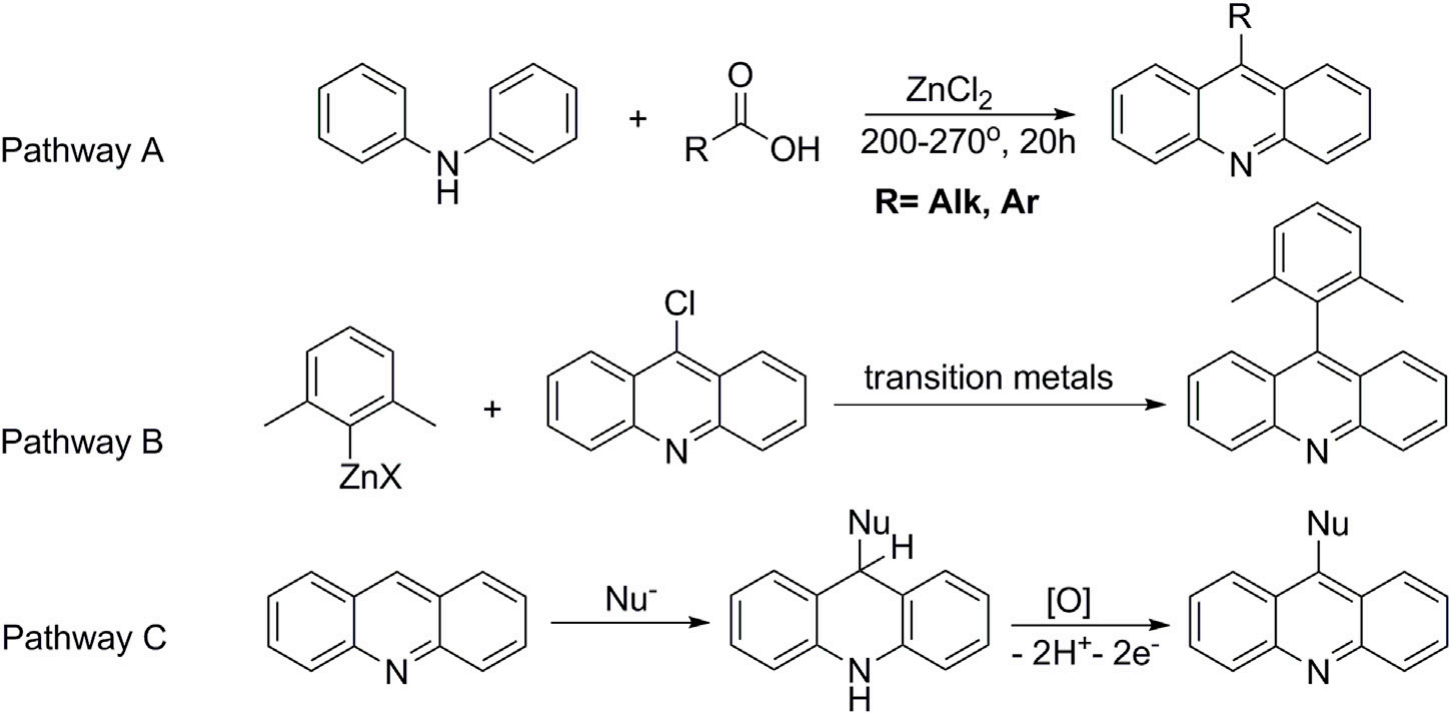
Methods for the preparation of 9-substituted acridines.

Currently, 9-substituted acridines can be obtained by several approaches. The classical method for the preparation of acridine derivatives is based on high-temperature condensation of diphenylamine and carboxylic acid in the presence of zinc chloride (Bernthsen, 1884) (Pathway A, Figure 2). Another method consists of preliminary functionalization of acridine and subsequent substitution of the introduced group by a nucleophile in the presence of transition-metal salts (Liu et al., 2013; Zhilyaev et al., 2022) (Pathway B, Figure 2). The presented strategies do not exclude a number of shortcomings. For example, the desire to introduce auxiliary groups, the use of catalysts and additional ligands, the employment of high temperature processes, and the need to separate reaction products from metal-containing byproducts complicate the preparation of target acridines. In addition, such accompanying negative factors as the high cost of the reaction process and, in some cases, low yields of the final products make the method inefficient and unprofitable. However, an alternative approach to functionalized acridines is nucleophilic aromatic substitution of hydrogen (S_N_^H^) (Chupakhin et al., 1994; Charushin and Chupakhin, 2005; Makosza, 2010; Mąkosza, 2011; Mąkosza and Wojciechowski, 2014). This method makes it possible to obtain 9-substituted acridines efficiently and directly, avoiding the disadvantages mentioned above.

The S_N_^H^ reactions proceed in two stages. The first one is nucleophilic addition to an aromatic substrate to form an intermediate (ϭ^H^-adduct). The ϭ^H^-adducts, *e.g.*, in the case of acridine, are stable compounds, which makes it possible to comprehensively study their physicochemical properties and biological activities (Chupakhin et al., 2017; Volkov et al., 2018). The second stage is characterized by an oxidative process with formation of the target-functionalized acridine (Pathway C, Figure 2).

We used the S_N_^H^ approach described in detail in a recent publication (Shchepochkin et al., 2021) to obtain the 9-phosphoryl-9,10-dihydroacridines **1a–f** and 9-phosphorylacridines **2a–f** studied in this work (Figure 1). Briefly, acridine and the corresponding phosphonate were stirred without solvent under an argon atmosphere at 75°C–80°C for 2–7 h. 9-Phosphoryl-9,10-dihydroacridines **1a-f** were isolated in 85%–95% yields. The corresponding aromatized compounds **2a-f** were obtained in 81%–89% yields by electrochemical oxidation of **1a–f**.

**FIGURE 3:**
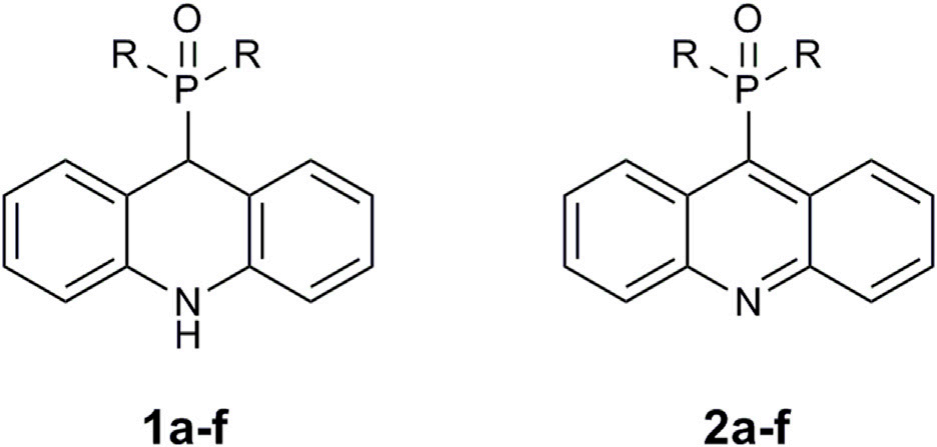
Structures of the studied acridine derivatives: 9-phosphoryl-9,10-dihydroacridines **1a–f** and 9-phosphorylacridines **2a–f**.

Figure 3 illustrates the structures of the previously described compounds, which were the subject of the research in the present work.

### 3.2 Inhibition studies of AChE, BChE and CES. Structure-activity relationships

Table 1 lists the components of the esterase profile of our compounds, i.e., their inhibitory potencies against the enzymes of cholinergic neurotransmission, AChE and BChE, and a structurally related off-target enzyme, CES (Makhaeva et al., 2016; Makhaeva et al., 2019b).

**TABLE 1:** Esterase profiles of the compounds and their inhibition of Aβ_42_ self-aggregation.

Compound	Phosphoryl substituent	Inhibitory activity against AChE, BChE and CES			Inhibition of Aβ_42_ self- aggregation, (%)^a^
		Human erythrocyte AChE, (%)^b^	Equine serum BChE, (%)^b^ or IC_50_ (µM)^c^	Porcine liver CES, (%)^b^	
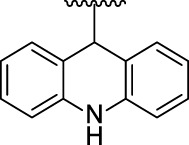 9-phosphoryl-9,10-dihydroacridines					
1a	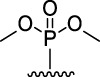	5.3 ± 1.3	21.3 ± 1.7	n.а	4.9 ± 0.4
1b	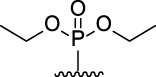	4.4 ± 1.1	16.9 ± 1.5	n.а	8.1 ± 0.8
1c	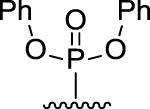	6.3 ± 0.9	48.0 ± 3.1 μM	21.6 ± 1.7	19.8 ± 1.5
1d	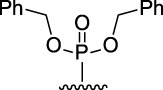	7.6 ± 1.2	**2.90 ± 0.23** μM	18.7 ± 1.4	58.9 ± 4.7
1e	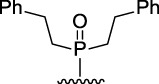	8.8 ± 2.0	**3.22 ± 0.25** μM	22.2 ± 1.7	46.9 ± 4.2
1f	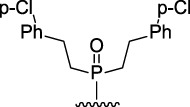	6.4 ± 1.0	21.7 ± 1.7 μM	11.4 ± 1.4	34.1 ± 2.7
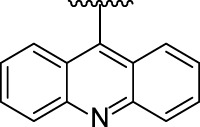 9-phosphorylacridines					
2a	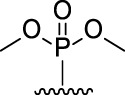	9.1 ± 1.2	9.0 ± 1.4	4.3 ± 1.1	4.9 ± 0.4
2b	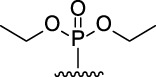	10.4 ± 1.2	10.8 ± 1.5	10.5 ± 1.3	n.а
2c	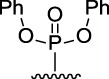	12.4 ± 1.4	32.7 ± 2.6	27.6 ± 2.2	3.6 ± 0.3
2d	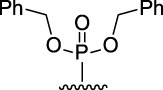	10.8 ± 1.5	**6.90 ± 0.55** μM	20.4 ± 1.8	n.а
2e	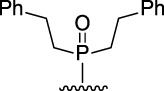	10.4 ± 1.3	25.7 ± 2.0	13.5 ± 1.7	n.а
2f	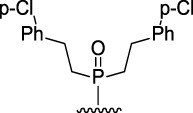	15.2 ± 1.3	20.4 ± 1.6	18.4 ± 1.4	11.2 ± 0.8
Tacrine		96.2 ± 0.2^d^	0.030 ± 0.002 μM	n.a	5.9 ± 0.5
BNPP		n.a	n.a	99.1 ± 0.93^e^	n.d
Myricetin		n.d	n.d	n.d	74.7 ± 5.2
Propidium iodide		n.d	n.d	n.d	90.7 ± 7.1

^b^ Compound concentration 20 µM.

^c^ Values without units of measurement for BChE inhibition correspond to % inhibition at 20 µM. For active compounds, BChE inhibition is presented as IC_50_ ± SEM μM, *n* = 3.

^d^ Tacrine IC_50_ AChE = 0.601 ± 0.047 µM.

^e^ BNPP IC_50_ CES = 1.80 ± 0.11 µM.

^a^ Inhibition of Aβ_42_ self-aggregation (50 µM) by the tested compound at 100 µM concentration. n. d.—not determined. n. a.—not active. Data are expressed as mean ± SEM, *n* = 3. IC_50_ BChE values for the most active compounds are shown in boldface font.

As can be seen from Table 1, all tested compounds weakly inhibited AChE, while a number of compounds demonstrated effective inhibition of BChE.

The most effective BChE inhibitors were found among the series of phosphorylated dihydroacridines. Moreover, the influence of the structure of the phosphorus-containing fragment on the inhibitory activity is clearly seen: dialkylphosphonates weakly inhibited BChE, but the introduction of aryl fragments increased the activity. Thus, diphenylphosphonate **1c** was a moderate inhibitor (IC_50_ = 48.0 ± 3.1 µM), while dibenzyloxy derivative **1d** and its diphenethyl bioisostere **1e** were effective inhibitors of BChE with IC_50_ = 2.90 ± 0.23 µM and 3.22 ± 0.25 µM, respectively. The introduction of a chlorine atom into the *para*-position of the phenethyl fragment of **1e** (compound **1f**) reduced tenfold the anti-BChE activity (IC_50_ = 21.7 ± 1.7 µM).

At the same time, aromatized analogs **2** of compounds **1** were largely inactive as BChE inhibitors. An exception was the acridine analog of the most effective dihydroacridine **1d**–dibenzyloxy derivative **2d**, which inhibited BChE with IC_50_ = 6.90 ± 0.55 µM, *i.e.*, it was only 2.4 times less effective than the corresponding dihydroacridine.

Weak inhibition of CES, an enzyme that catalyzes the hydrolysis of numerous ester-containing drugs, is a positive property of the studied compounds because this decreases the likelihood of unwanted drug-drug interactions in the event of their use in AD therapy (Tsurkan et al., 2013; Makhaeva et al., 2019b).

### 3.3 Kinetic studies of BChE inhibition

**FIGURE 4:**
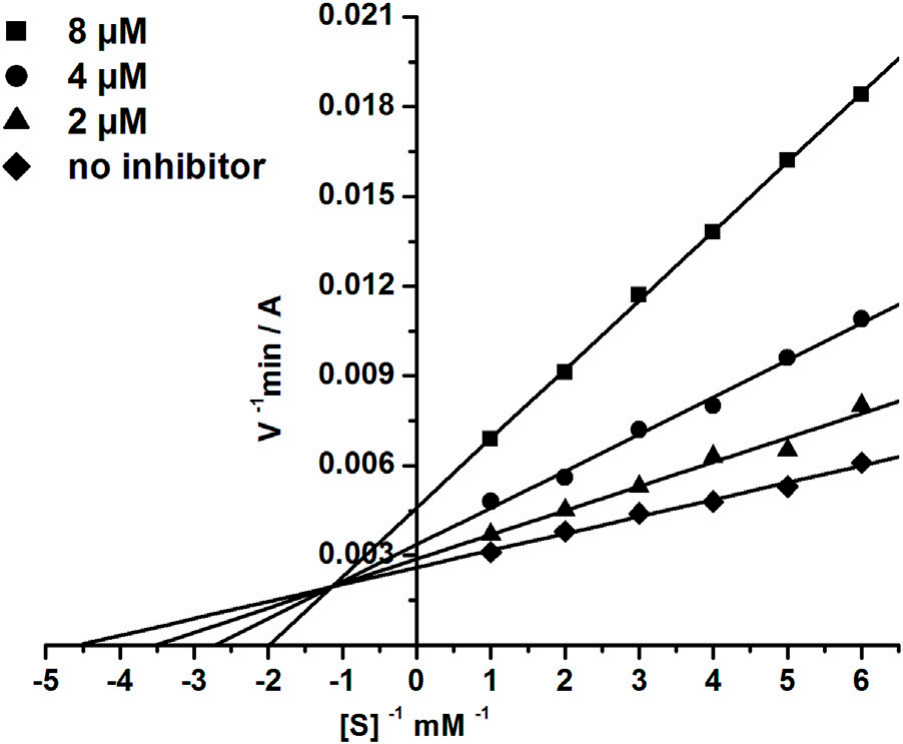
Double-reciprocal (Lineweaver-Burk) plots of the steady state inhibition of BChE by compound **1d**.

The most active BChE inhibitor **1d** was selected for inhibition kinetics studies. Lineweaver-Burk plots-double reciprocal representations of Michaelis-Menten parameters-were used to assess the type of inhibition. Graphical analysis of the kinetic data on BChE inhibition by the tested compound (Figure 4) demonstrates changes in both *K*_m_ and *V*_max_ values; this result attests to a mixed type of inhibition. The values obtained for the competitive component (*K*_i_) and the non-competitive component (α*K*_i_) of the constants of BChE inhibition by compound **1d** were 2.22 ± 0.19 µM and 11.5 ± 0.9 µM, respectively.

### 3.4 Molecular docking to AChE and BChE

To investigate the structural origins of the observed differences in inhibitory activity, we performed molecular docking to AChE and BChE for the most active dihydroacridine derivatives, namely, the bioisosteric compounds **1d** and **1e** and their aromatized analogs **2d** and **2e.**

**FIGURE 5:**
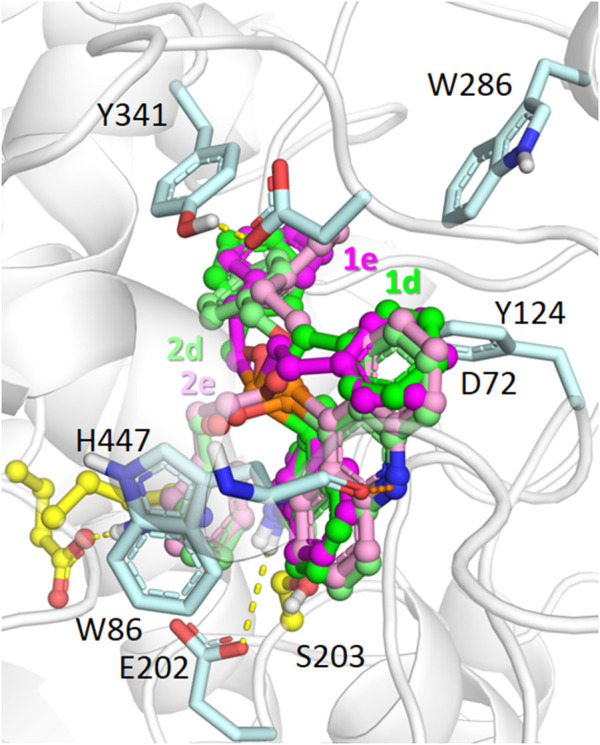
Binding modes of compounds **1d,e** (carbon atoms colored green/magenta, respectively) and **2d**,**e** (carbon atoms colored with lighter shades of the same colors) in the active site gorge of human AChE (PDB: 4EY7), according to molecular docking results. Yellow dashes show direct hydrogen bonding interactions. Orange dashes show the hypothetical possibility of water molecule-mediated interactions.

According to the results of molecular docking, compounds with benzyloxy and phenethyl substituents **1d**,**e**, and **2d**,**e** were able to bind to the AChE active site gorge in a similar manner (Figure 5). However, in this case, the bulky dihydroacridine/acridine and benzyloxy/phenethyl groups were located below amino acid residues Tyr341 and Tyr124, which are the part of a narrow bottleneck (Sussman et al., 1991; Wlodek et al., 1997). This makes trafficking of the ligands to the bottom of the gorge kinetically hindered (Lushchekina and Masson, 2020), thus reducing their inhibitory efficacy.

**FIGURE 6:**
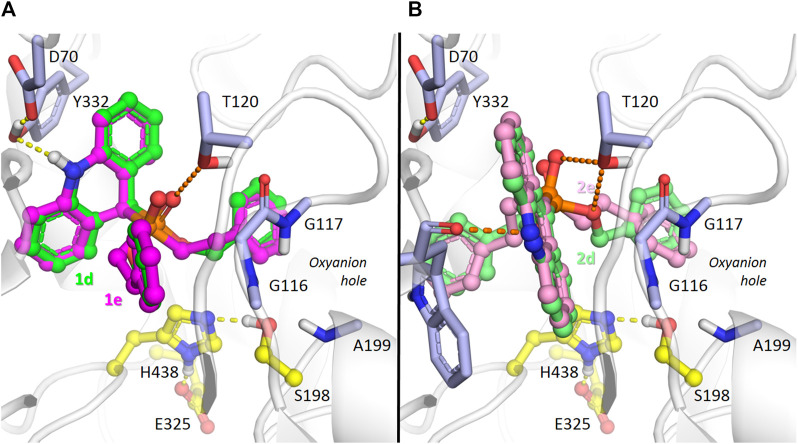
Binding mode of compounds **1d**,**e** (carbon atoms colored green or magenta, respectively) **(A)**, and **2d**,**e** (carbon atoms colored with lighter shades of the same colors) **(B)** in the active site gorge of human BChE (PDB: 1P0I), according to molecular docking results. Yellow dashes show direct hydrogen bonding interactions. Orange dashes show the hypothetical possibility of water molecule-mediated interactions.

With respect to BChE docking (Figure 6), a significant difference of binding to the enzyme active site at the bottom of the gorge was observed between dihydroacridine/acridine compounds **1d**,**e** and **2d**,**e** with benzyloxy/phenethyl substituents. Due to the wider active site gorge of BChE compared to AChE (Saxena et al., 1997), no kinetic difficulties of such binding were expected.

According to the docking results, the dihydroacridine derivatives **1d**,**e** form direct hydrogen bonds with the Tyr332 and D70 side chains (Figure 6A). There is also a theoretical possibility of interaction of the phosphoryl group with the Thr120 side chain oxygen atom through a bridging water molecule (Figure 6A). According to X-ray crystallography (Koellner et al., 2000; Bourne et al., 2010; Berg et al., 2011), and molecular modeling (Nascimento et al., 2017; Makhaeva et al., 2021; van der Westhuizen et al., 2022), such interactions are typical for protein-ligand interactions in the active site gorge of cholinesterases.

Regarding acridines **2d** and **2e**, no specific interactions were observed, which agrees with their lower experimentally observed inhibitory activity. However, interactions of the acridine fragment of both compounds with the Trp82 main chain oxygen atom are possible (Figure 6B). We observed such a water-mediated interaction for the similar amiridine fragment and the homologous AChE residue Trp86 in molecular dynamics simulations (Makhaeva et al., 2021). As observed with dihydroacridine derivatives, the phosphoryl group of the acridine compounds was located in such a way to suggest that a similar water molecule-bridged interaction occurred with the oxygen atom of the Thr120 side chain (Figure 6B). Regarding the dibenzyloxy group of compound **2d**, such interactions are also possible for the oxygen atom (Figure 6B), which is consistent with its higher inhibitory activity compared to compound **2e** (Table 1).

### 3.5 Molecular dynamics simulations of the complex of compound 1d with BChE

**FIGURE 7:**
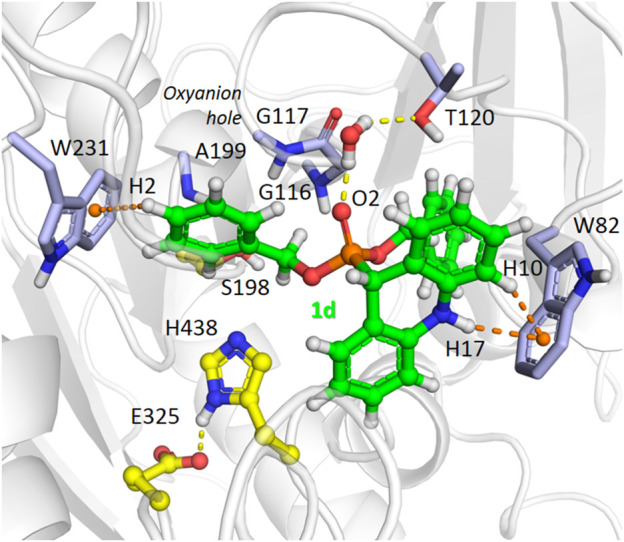
A snapshot from the MD simulation of the complex of compound **1d** with BChE, showing the main interactions in the complex after the ligand reorientation discussed in the text. Yellow dashed lines indicate hydrogen bonds, and orange dashes show stacking interactions. The ligand’s atoms referred to in the following figure are labeled. Note the presence of a bridging water molecule whose hydrogen atoms form hydrogen bonds with the Tyr120 O^γ^ atom and the ligand O2 atom.

**FIGURE 8:**
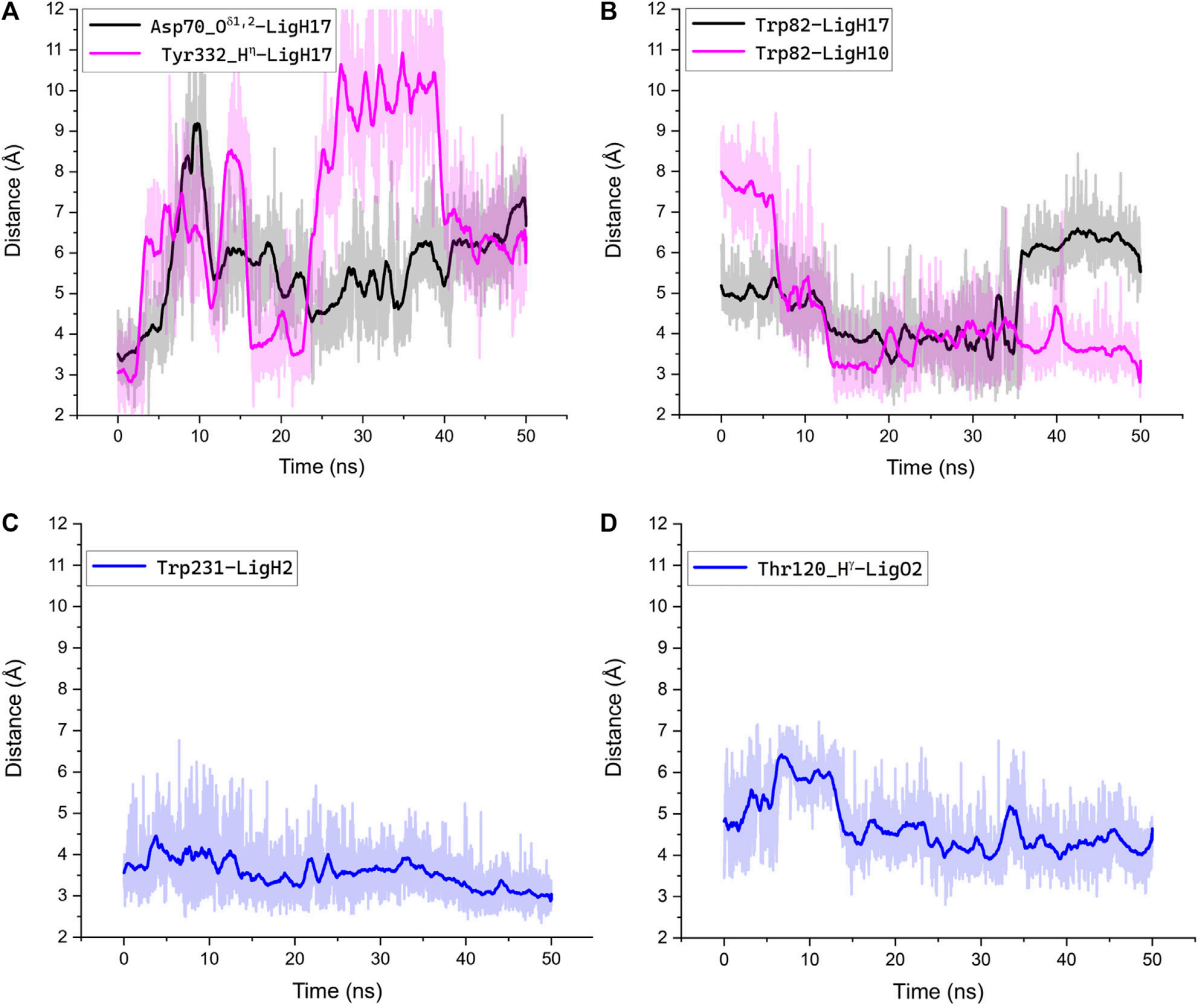
Interactions along the MD trajectory of the complex of compound **1d** with BChE. The evolution of distances between specified atoms or center of masses for the indole rings of Trp residues during the MD simulation is shown. The ligand atoms are indicated according to the labeling in Figure 7. Bold lines show distances averaged over 1 ns of the simulation, and the light trace shadow shows distances recorded every 10 ps. **(A)** The increase of the shown distances reflects the disruption of interactions between the dihydroacridine group of the ligand and Asp70 and Tyr332 residues and following reorientation; **(B)** the decrease of the shown distances reflects the establishment of new interactions between the dihydroacridine group of the ligand and the side chain of Trp82 after the ligand reorientation; **(C)** T-stacking interaction of one of the ligand’s phenyl rings with the Trp231 indole ring was maintained through the whole trajectory and tended to be tighter by the end of the simulation; **(D)** after the ligand reorientation, the phosphoryl oxygen atom remained close enough to the Thr120 hydroxy group for indirect interactions via bridging water molecules. Only occasionally was the distance between them less than 3Å, which suggests the possibility of transient direct interactions in the form of weak hydrogen bonds.

**FIGURE 9:**
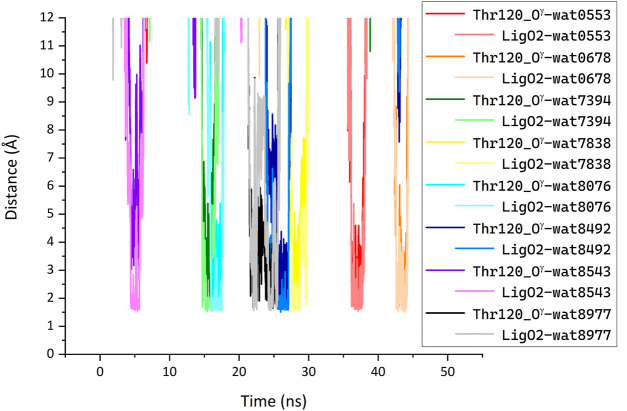
Bridging of the ligand phosphate group and Thr120 side chain by water molecules (LigO2···H^1/2^-O-H^1/2^·· Thr120_O^γ^) identified in the MD simulation of the complex between compound **1d** with BChE. Each line shows the minimum of the two distances between each of the designated water molecule hydrogen atoms and enzyme or ligand oxygen atom. A water molecule was considered bridging when such H-O distances were less than 2 Å simultaneously for the ligand O2 and the enzyme Thr120_O^γ^ atoms; pairs of these distances for the same water molecule are shown with different shades of the same color. Along the MD trajectory, 133 different water molecules participated in bridging defined in such a way. The plot shows distances for a few water molecules that were most frequently identified as bridging. For clarity, distances for the other type of bridging (LigO2···H^1/2^-O·· Thr120_H^γ^) are not shown.

We performed MD simulations for the complex of the most active compound **1d** with BChE. During the MD trajectory, the ligand remained bound to the enzyme, though it had reoriented, and the resulting binding pose (Figure 7) differed from the docked one (Figure 6A) in specific details. While molecular docking showed that the dihydroacridine group interacted with side chains of the PAS residues Asp70 and Tyr332, these interactions were disrupted shortly after the start of the MD simulation (Figure 8A). Instead, the inhibitor reoriented so that the dihydroacridine group established new interactions with the side chain of Trp82 after 15 ns of simulation (Figure 8B). This residue is located deeper in the gorge than Asp70 and Tyr332 and forms the active site as a part of the cation-binding compartment. The newly established π-cation stacking or T-stacking interactions of the dihydroacridine fragment were maintained during the remainder of the MD trajectory. In addition, T-stacking interactions of one of the ligand’s phenyl rings with the Trp231 indole ring were observed during the entire MD trajectory (Figure 8C). The reorientation of the ligand in the active site gorge of BChE took place during the first 15 ns of the simulation and included temporary displacement of the phosphate group. After this, the phosphate group returned to the position outside the oxyanion hole and close to the Thr120 side chain (Figure 7; Figure 8D). The MD simulation confirmed that their interaction was maintained mainly through frequently exchanging bridging water molecules (Figure 9). However, occasional direct hydrogen bonding is also possible due to thermal motion (Figure 8D).

### 3.6 Inhibition of β-amyloid (1-42) (Aβ_42_) self-aggregation

The *in vitro* inhibitory activity of the test compounds against Aβ_42_ self-aggregation was determined using the thioflavin T (ThT) fluorimetric assay (LeVine, 1999; Bartolini et al., 2003; Munoz-Ruiz et al., 2005; Bartolini et al., 2011). This widely used procedure is based on specific binding of the fluorescent dye ThT to the β-sheets of assembled amyloid fibrils leading to a significant increase in fluorescence signal (Biancalana and Koide, 2010). Therefore, the decrease in ThT fluorescence in the presence of the studied compound correlates with its activity to inhibit the formation of amyloid aggregates.

As can be seen from Table 1, the ability of the studied compounds to inhibit the self-aggregation of Aβ_42_ depends both on the structure of their acridine moiety and the substituents at the phosphorus atom; moreover, the structure-activity relationship largely coincides with that observed for BChE inhibition.

Indeed, acridine derivatives **2** inhibit the self-aggregation of Aβ_42_ very weakly or do not inhibit at all. The ability of dihydroacridines **1** to block Aβ_42_ self-aggregation depends on the structure of the phosphoryl fragment: dialkoxy derivatives are weak inhibitors, but the introduction of aryl substituents increases activity, which increases upon going from diphenoxy (**1с**) to dibenzyloxy (**1d**) substituents. Compound **1d** exhibits the maximum inhibitory activity and suppresses the formation of amyloid aggregates by 58.9% ± 4.7%. Its bioisosteric analog, the diphenethyl derivative **1e**, also has high activity, inhibiting Aβ_42_ self-aggregation by 46.9% ± 4.2%. Adding a chlorine atom to the *para*-position of the phenethyl fragment **1e** (compound **1f**) slightly reduces the inhibitor activity.

### 3.7 Molecular docking to Aβ_42_

Molecular docking did not reveal a direct explanation of the experimentally observed differences in the ability of acridine/dihydroacridine compounds to inhibit Aβ_42_ self-aggregation. There were slight differences in estimated binding energies (Supplementary Table S1) and number of contacts (Supplementary Table S2) of dihydroacridine derivatives **1d**,**e** compared to acridine derivatives **2d**,**e** with Aβ_42_ conformers obtained from the NMR structure (PDB: 1IYT) (Crescenzi et al., 2002) [see details in (Makhaeva et al., 2022)]. Although these differences could be considered minor, they are in line with different conformations of the Aβ_42_ peptide used as molecular docking targets.

Another plausible reason for the experimentally observed substantial differences between active dihydroacridine inhibitors and practically inactive acridine compounds could be related to the different ability of planar aromatic acridines to self-associate through π-π stacking interactions compared to non-planar dihydroacridines. Such self-stacking of acridine derivatives is well known (Evstigneev et al., 2006; Franco Pinto et al., 2022) and could compete with binding to the Aβ_42_ peptide (Gao et al., 1991; Buurma and Haq, 2008).

### 3.8 Antioxidant activity (AOA)

Table 2 lists the results of our determinations of the AOA of compounds **1a–f** and **2a–f** using the ABTS and FRAP assays. In the ABTS test, the radical cation ABTS^•+^ reacts with an antioxidant via one or both of the following pathways: single-electron transfer (SET) or hydrogen-atom transfer (HAT). In the FRAP test, the ferric 2,4,6-tripyridyl-s-triazine complex [Fe (TPTZ)_2_]^3+^ is reduced to the corresponding ferrous complex [Fe (TPTZ)_2_]^2+^ by the SET mechanism. In these tests, we used Trolox and ascorbic acid as the reference and positive control compounds, respectively.

**TABLE 2:** Antioxidant activity (AOA) of compounds **1a–f** and **2a–f** in the ABTS and FRAP tests.

Compound	Phosphoryl substituent	ABTS^•+^-scavenging activity		FRAP Fe^3+^-reducing activity
		TEAC ^a^	IC_50_, μM ^b^	TE ^a^
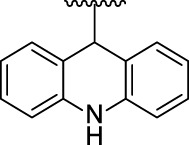 9-phosphoryl-9,10-dihydroacridines				
1a	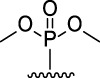	0.99 ± 0.04	21.3 ± 1.4	1.60 ± 0.01
1b	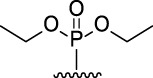	0.97 ± 0.03	20.8 ± 1.3	1.15 ± 0.01
1c	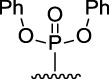	1.13 ± 0.05	17.8 ± 1.1	1.13 ± 0.02
1d	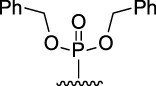	0.97 ± 0.04	20.8 ± 1.3	0.97 ± 0.06
1e	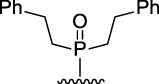	0.90 ± 0.03	21.8 ± 0.9	1.10 ± 0.01
1f	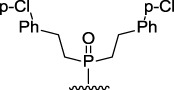	0.80 ± 0.03	22.6 ± 0.7	0.81 ± 0.02
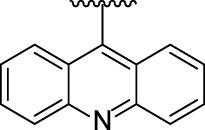 9-phosphorylacridines				
2a	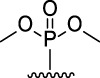	n.a	n.d	0.08 ± 0.02
2b	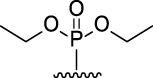	n.a	n.d	0.07 ± 0.01
2c	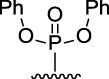	n.a	n.d	n.a
2d	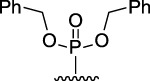	n.a	n.d	n.a
2e	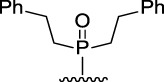	n.a	n.d	n.a
2f	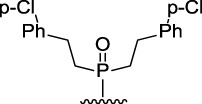	n.a	n.d	n.a
Trolox		1.0	20.1 ± 0.7	1.0
Ascorbic acid		0.97 ± 0.04	22.4 ± 2.2	1.23 ± 0.11

^a^ TEAC, Trolox equivalent antioxidant capacity (ABTS) and TE–AOA, in Trolox equivalents (FRAP) (for the calculations, see Materials and methods).

^b^ Compound concentration required for a 50% reduction in the concentration of the ABTS radical cation.

n.d. - not determined. n.a. - not active.

All derivatives of 9,10-dihydroacridine **1** demonstrated high radical-scavenging activity in the ABTS test at levels near or slightly above that of Trolox, the standard antioxidant. In contrast, the 9-phosphoryl-acridines **2** were not active in the ABTS test. It should also be noted that compounds **1a–d** exhibited a high initial rate of the ABTS^•+^ binding reaction, comparable to Trolox (the maximum degree of radical binding was achieved within 1 min). This fact is consistent with the SET mechanism for the antiradical activity of these compounds. A slight decrease in the radical-scavenging activity was observed for the phenethyl derivative **1e** and its *para*-Cl-substituted analog **1f**, which was also accompanied by a decrease in the reaction rate (the time to reach the maximum degree of radical binding increased from 1 to 30 min).

All dihydroacridine derivatives **1a–f** also showed high iron-reducing activity in the FRAP test, at the level or above that of Trolox. Moreover, these compounds were relatively rapid antioxidants, reaching the maximum effect within 4 min. The high activity of dihydroacridines **1a–f** in the Fe^3+^-reducing test is probably directly related to the ease of their electrochemical oxidation to acridines (Shchepochkin et al., 2021). The dimethoxy derivative **1a** (1.60 ± 0.01 TE) was the lead compound in this series, and the least active compound was *para*-Cl-substituted diphenethylphosphoryl dihydroacridine **1f** (0.81 ± 0.02 TE). In general, the results of the ABTS and FRAP tests were in good agreement with each other.

Phosphoryl acridines **2a–f** had almost no activity in the FRAP test. Only dimethoxy and diethoxy derivatives **2a,b**, which are the aromatic analogs of the most active compounds **1a,b** in the dihydroacridine series, showed detectable activity.

### 3.9 Quantum-chemical calculations of AOA

The FRAP assay is a typical SET method (Gulcin, 2020; Spiegel et al., 2020; Florin Danet, 2021), where [Fe (TPTZ)_2_]^3+^ is reduced to [Fe (TPTZ)_2_]^2+^ by electron transfer from an antioxidant molecule. Unlike [Fe (TPTZ)_2_]^3+^, ABTS^•+^ can be reduced by acquiring both a hydrogen atom and an electron, depending on the antioxidant structure, solvent type, and pH (Ilyasov et al., 2020). In particular, SET is facilitated at acidic pH (Gulcin, 2020). In the ABTS method used in this work, the main solvent is ethanol at pH∼4.5. Under these conditions, the ABTS^•+^ molecule is deprotonated at both SO_3_ groups; it is reduced by acquiring an electron from the antioxidant, similarly to [Fe (TPTZ)_2_]^3+^ (Ivanova et al., 2020).

In both methods, reduction can occur by either the direct SET mechanism characterized by the ionization potential (IP), or by a hydrogen atom abstraction from the antioxidant molecule, when an electron is transferred to the radical and a proton to a solvent molecule. In the latter case, the actual antioxidant mechanism may be a complex multistage process, but the overall reaction can be characterized by the enthalpy of a hydrogen atom abstraction*, i.e*., the bond dissociation enthalpy, also known as the bond dissociation energy (BDE).

The quantum mechanical (QM) characteristics of possible antioxidant reactions for dihydroacridines and acridines were calculated taking two items into account: 1) the protonation state of the investigated structures in water and ethanol for the FRAP and ABTS tests, respectively; and 2) the method of synthesizing acridines from dihydroacridines by two-electron transfer electrochemical oxidation (Shchepochkin et al., 2021). The second item suggested a two-stage mechanism for the interaction of the antioxidant with ABTS^•+^ or FRAP molecules.

The calculated IP and BDE values for acridines were much higher than the corresponding values for dihydroacridines. Moreover, the characteristics of acridines were close to each other, as were the characteristics of dihydroacridines, which agrees with similar experimental results for compounds **2a** and **1a**, respectively. A detailed description of the approach that was used and all calculated characteristics are given in Supplementary Materials. Here, we briefly summarize the results.

The obtained QM characteristics for the two-stage mechanism of interaction of the antioxidant and ABTS^•+^ or FRAP molecules allowed us to suggest that each dihydroacridine molecule donates two electrons, yielding the corresponding acridine molecule, and thereby reducing two molecules of ABTS^•+^ or FRAP. At the same time, the reduction of the second radical molecule was characterized by low values of the BDE, which was consistent with the high AOA of dihydroacridines in both tests. The poor AOA of acridines agrees with high values of their QM characteristics.

**FIGURE 10:**
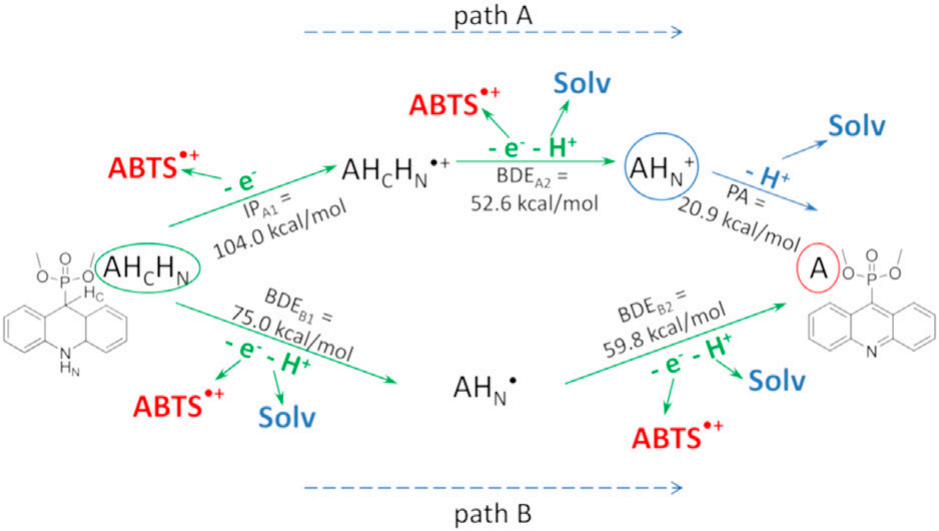
Two-stage mechanism of the antioxidant action of dihydroacridines in the ABTS test using compound **1a** as an example. The parent dihydroacridine **1a** (AH_C_H_N_) is circled in green, the final acridine **2a** (A) is circled in red, and the protonated form of acridine (AH_N_^+^) is circled in blue. The calculated QM characteristics of each stage are shown along the corresponding arrows indicating the reaction. “Solv” is the solvent molecule.

The suggested two-stage mechanism of the dihydroacridine antioxidant action is illustrated in Figure 10 for compound **1a** in the ABTS test as an example. In path A, the antioxidant (dihydroacridine, AH_C_H_N_) sequentially loses an electron and a hydrogen atom to generate the protonated form of the corresponding acridine (AH_N_^+^). The latter dissociates to acridine (A); this process is characterized by the proton affinity (PA). In path B, the antioxidant sequentially loses two H-atoms to form the corresponding acridine.

In both cases, the reaction enthalpy of the second stage was low, indicating that the reduction of the second ABTS^•+^ molecule occurred immediately after the reduction of the first one. For comparison, the IP and the enthalpy of a hydrogen atom abstraction of the phenol group of Trolox, calculated in ethanol, were 98.0 and 73.0 kcal/mol, respectively.

A noticeably higher AOA of compound **1a** with methoxy substituents compared to other compounds **1** in the FRAP test and the similar AOA in the entire series of compounds **1** in the ABTS test could be explained by the different access of antioxidant molecules to the ABTS^•+^ radical *versus* the [Fe (TPTZ)_2_]^3+^ cation. Indeed, [Fe (TPTZ)_2_]^3+^, with a three-dimensional propeller structure, is bulkier than the quasi-one-dimensional structure of ABTS^•+^ (Supplementary Figure S1), which hinders access for bulky antioxidants.

### 3.10 Prediction of ADMET, physicochemical, and PAINS profiles

Table 3 shows the predicted ADMET properties and physico-chemical characteristics that we computed for compounds **1a–f** and **2a–f**. As can be seen, with the exception of the phenethyl and chlorophenethyl derivatives **1e–f** and **2e–f**, we obtained favorable values for intestinal absorption, indicating that the compounds would be expected to be bioavailable when administered orally. Note that it is possible that the unfavorable numbers for compounds **1e–f** and **2e–f** were likely due to their being outside of the applicability domain of the model. Given the high BBB permeability values, the compounds would be expected to enter the brain to exert effects on CNS targets; this is especially so for the benzyl and phenethyl derivatives. Parameters for cardiac toxicity risk, hERG p*K*_*i*_ and pIC_50_, were within 5.0–7.9 log units, near the center of the possible range of 3–9 log units. Lipophilicities, as assessed by computed LogP values, were in some cases outside of the classical Lipinsky rule of 5 range, but these values were deemed unreliable owing to their being outside of the applicability domain of the model. Otherwise, the molecular weights and predicted aqueous solubility values were near or within acceptable limits for drug-like molecules. Integral quantitative estimates of drug likeness (QED) fell within a range of 0.2–0.9; the most active compounds, **1d,e**, scored 0.3 for this parameter. Finally, no alerts were identified by the Pan Assay INterference compoundS (PAINS) filter.

**TABLE 3:** Predicted ADMET and physicochemical profiles of compounds **1a–f, 2a–f**.

Compound	MW	LogP_ow_	pS_aq_	LogBB	HIA, %	hERG *pK*_*i*_	hERG *pIC*_*50*_	QED
1a	289.27	2.66	2.55	0.08	75	4.97	5.15	0.85
1b	317.32	3.54	3.32	0.08	90	5.24	5.28	0.78
1c	413.41	5.13	5.57	0.08	75	6.05	6.69	0.35
1d	441.47	4.85	5.02	0.49	75	5.98	6.38	0.30
1e	437.52	6.09	6.10	0.62	15	5.92	6.71	0.30
1f	506.41	6.88	6.71	0.21	15	5.83	7.56	0.25
2a	287.25	2.22	2.21	0.21	70	5.46	5.30	0.55
2b	315.31	2.89	3.14	0.21	86	5.72	5.41	0.52
2c	411.40	4.83	5.80	0.21	70	6.52	6.65	0.25
2d	439.45	4.37	5.34	0.60	70	6.44	6.38	0.21
2e	435.51	5.89	6.15	0.71	11	6.38	6.68	0.21
2f	504.40	6.70	6.80	0.33	11	7.07	7.87	0.17
Tacrine	198.27	2.95	1.52	0.00	93	4.98	4.98	0.71

MW, molecular weight; LogP_ow_, octanol-water partition coefficient; pS_aq_, aqueous solubility [−log(M)]; LogBB, blood–brain barrier distribution; HIA, human intestinal absorption [%], hERG *pKi*—hERG, potassium channel affinity [−log(M)], hERG *pIC*_*50*_—hERG, potassium channel inhibitory activity [−log(M)]; QED, quantitative estimate of drug-likeness.

Taken together, the results of the computed ADMET, physico-chemical, and PAINS profiles indicate that the lead compounds were acceptable for being advanced to further optimization and experimental studies of efficacy and safety.

## 4 Conclusion

Thus, studying the esterase profile of two new groups of acridine derivatives, 9-phosphoryl-9,10-dihydroacridines **1** and 9-phosphoryl-acridines **2**, showed that all new compounds weakly inhibited AChE. Dihydroacridines with aryl substituents in the phosphoryl fragment inhibited BChE, with dibenzyloxy derivative **1d** and its diphenethyl bioisostere **1e** being the most active**.** Among acridines **2**, only one compound **2d,** an aromatized analog of **1d,** proved to be an effective BChE inhibitor. Using 9-bis(benzyloxy)phosphoryl-9,10-dihydroacridine **1d** as an example, a mixed mechanism of BChE inhibition was demonstrated from a kinetics analysis. The results of molecular docking served to explain the experimentally determined parameters of the potency, selectivity, and mechanism of cholinesterases inhibition by the acridine derivatives. Weak inhibition of CES, an off-target esterase, is a positive characteristic of the studied compounds, because this reduces the likelihood of unwanted drug-drug interactions in the case of their use in AD therapy.

The ability of the studied acridine derivatives to inhibit the self-aggregation of Aβ_42_ depended on both the structure of the acridine moiety and the structure of the substituents at the phosphorus atom. Moreover, the structure-activity relationship largely coincided with that observed for BChE inhibition. Dihydroacridines **1**, especially with aryl substituents in the phosphoryl moiety, were able to block Aβ_42_ self-aggregation. The most active were the dibenzyloxy derivative **1d** and its bioisosteric analog, the diphenethyl derivative **1e**. On the other hand, acridine derivatives **2** inhibited Aβ_42_ self-aggregation weakly or not at all. Such a difference may be connected to the different ability to self-associate through π-π stacking interactions of the planar aromatic structure of acridines compared to non-planar dihydroacridines, which could compete with their binding to the Aβ_42_ peptide.

All 9-phosphoryl-9,10-dihydroacridines **1** demonstrated high ABTS^•+^-scavenging and iron-reducing activity at the level of Trolox, unlike 9-phosphoryl-acridines **2,** which were not active. Quantum-chemical calculations confirmed this difference.

Calculated ADMET parameters of the test compounds predicted favorable intestinal absorption, good blood–brain barrier permeability, and cardiac toxicity potential.

In summary, compounds **1d** and **1e** with dibenzyloxy- and diphenethyl- substituents were found to display high inhibition of BChE activity and Aβ_42_ self-aggregation, high antioxidant capability, and favorable predicted ADMET properties. Therefore, these molecules can be considered lead compounds and are recommended for further in-depth studies as promising anti-AD agents.

## Data Availability

The original contributions presented in the study are included in the article/Supplementary Material. Further inquiries can be directed to the corresponding author.
