# Supplementary material for: Derivatives of 9-phosphorylated acridine as butyrylcholinesterase inhibitors with antioxidant activity and the ability to inhibit β-amyloid self-aggregation: potential therapeutic agents for Alzheimer’s disease
Source: Front Pharmacol. 2023 Aug 9;14:1219980. doi: 10.3389/fphar.2023.1219980 (PMC10466253; doi:10.3389/fphar.2023.1219980)
Supplement: Supplementary file 1 [file DataSheet1.docx]

Supplementary Material

Derivatives of 9-Phosphorylated Acridine as Butyrylcholinesterase Inhibitors with Antioxidant Activity and the Ability to Inhibit β-Amyloid Self-Aggregation: Potential Therapeutic Agents for Alzheimer’s Disease

Galina F. Makhaeva, Nadezhda V. Kovaleva, Elena V. Rudakova, Natalia P. Boltneva, Sofya V. Lushchekina, Tatiana Yu. Astakhova, Elena N. Timokhina, Olga G. Serebryakova, Alexander V. Shchepochkin, Maxim A. Averkov, Irina A. Utepova, Nadezhda S. Demina, Eugene V. Radchenko, Vladimir A. Palyulin, Vladimir P. Fisenko, Sergey O. Bachurin, Oleg N. Chupakhin, Valery N. Charushin, and Rudy J. Richardson*

*** Correspondence:** Rudy J. Richardson: rjrich@umich.edu

# Supplementary Data

## Quantum-chemical calculations of AOA

When evaluating the AOA of the studied compounds, the protonation state was taken into account. The pKa values were estimated by the Calculator Plugins of Marvin 21.14.0, ChemAxon (<https://www.chemaxon.com>, accessed on 27 January 2023) and free MolGpKa (Pan et al., 2021). The acridines pKa values predicted by Marvin software and MolGpKa were 4.7 and 4.0, respectively. The dihydroacridines pKa values predicted by Marvin software and MolGpKa were -1.0 and 3.1, respectively (which agrees with the known pKa of diphenyl amine). This means that in the FRAP test with pH 3.6, acridines are protonated and dihydroacridines are neutral.

In the ABTS test, the main solvent is ethanol at pH ~4.5. Under these conditions both dihydroacridines and acridines are neutral. It is known that the pKa value of pyridine decreases from 5.152 in water to 3.809 in a 30:70 (v/v) H_2_O:ethanol mixture (Gowland and Schmid, 1969). We assumed that the pKa of acridines also significantly decreased with an increase of the ethanol fraction in the water/ethanol mixture, and the pKa value of acridines in the ABTS test was less than 4.0 (ethanol fraction was more than 80% by volume). Then, under these conditions, acridine molecules would be mostly neutral.

In the following, dihydroacridines are referred to as AH_C_H_N_ and acridines are referred to as A.

## AOA of dihydroacridines and acridines in the ABTS test

The suggested mechanism for antioxidant reactions of dihydroacridines and acridines in the ABTS test is presented below (Scheme S1).

The two-stage antioxidant mechanism of dihydroacridines can be implemented in two ways, marked by path A and path B in the scheme. In path A, the antioxidant donates an electron to the first radical at the first stage A1. This reaction is characterized by the enthalpy of the electron abstraction from neutral dihydroacridine AH_C_H_N_, IP_A1_. At the second stage of path A, the resulting dihydroacridine radical cation AH_C_H_N_^•+^ loses the H atom, while the electron passes to the second radical, and the proton passes to the solvent molecule. This stage is characterized by a low enthalpy of the H-atom abstraction from AH_C_H_N_^•+^, BDE_A2_. As a result of these two steps, the protonated form of the corresponding acridine is formed: AH_N_^•+^. Then, AH_N_^•+^ further deprotonates, as acridines are neutral in ethanol under experimental conditions. Deprotonation is characterized by the proton affinity (PA) calculated as follows.

| PA = *H*(A) + *H*(H^+^) – *H*(AH^+^), | (S1) |
| --- | --- |

where *H*(A) is the enthalpy of the acridine molecule; *H*(AH^+^) is the enthalpy of the protonated acridine molecule; and *H*(H^+^) is the proton enthalpy.

In path B, the dihydroacridine molecule loses H_C_ at the first stage B1, which is characterized by BDE_B1_ of the neutral molecule AH_C_H_N_. At the second stage B2, the AH_N_^•^ radical formed during the first stage loses a H_N_ atom. This stage is characterized by the enthalpy of the H-atom abstraction from AH_N_^•^, BDE_B2_. As a result of the two stages of path B, the corresponding neutral acridine is formed.

A consequence of the two-stage antioxidant mechanism is that the corresponding neutral acridine molecule is formed from the parent dihydroacridine molecule. The IP values of acridines calculated in ethanol are high, which explains their low AOA.

The QM characteristics calculated in ethanol solvent at each stage of both paths are presented in Table S1. IP and BDE values for all reactions in Scheme S1 were calculated as follows:

| IP_A1_ = *H*(AH_C_H_N_^•+^) + *H*(e^-^) – *H*(AH_C_H_N_) | (S2) |
| --- | --- |
| BDE_B1_ = *H*(AH_N_^•^) + *H*(H^•^) – *H*(AH_C_H_N_) | (S3) |
| BDE_A2_ = *H*(AH_N_^+^) + *H*(H^•^) –- *H*(AH_C_H_N_^•+^) | (S4) |
| IP^a^ = *H*(A^•+^) + *H*(e^-^) – *H*(A), | (S5) |

where:

*H*(AH_C_H_N_) is the enthalpy of the dihydroacridine molecule; *H*(AH_C_H_N_^•+^) is the enthalpy of the dihydroacridine radical cation after the electron abstraction; *H*(e^-^) is the electron enthalpy; *H*(AH_N_^•^) is the enthalpy of the antioxidant radical after H_C_ atom abstraction from AH_C_H_N_; *H*(AH_N_^+^) is the enthalpy of the antioxidant cation after sequential electron and H_C_ atom abstractions from AH_C_H_N_; *H*(A) is the enthalpy of the acridine molecule obtained after sequential H_C_ and H_N_ abstractions from AH_C_H_N_; *H*(H^•^) is the H-atom enthalpy; *H*(A^•+^) is the enthalpy of the acridine radical cation after the electron abstraction.

The enthalpy of the proton and solvated electron were calculated in the same way as in Ref. (Marković et al., 2016). For acridines in ethanol, only IP values were calculated as they do not have reactive hydrogen atoms.

Based on the calculated reaction enthalpies, we cannot choose the preferred antioxidant pathway for dihydroacridines. To do this, it is necessary to calculate the change in reaction entropy, taking into account all the reactants, including the reduced radical, counterions, and a solvent molecule taking on the proton, which is beyond the scope of this work. In addition, in charge transfer between an electron donor and an electron acceptor, the configurational arrangement of interacting molecules is very important (Park et al., 2017), which also affects the observed activity.

It is likely that both ways are implemented in proportion. However, based on the calculated characteristics, we can say that the difference in AOA between dihydroacridines and acridines is due to the significantly higher energy costs in the antioxidant reaction in the case of acridines as opposed to dihydroacridines.

## FRAP AOA of dihydroacridines and acridines

In the FRAP test, the general picture of dihydroacridines and acridines AOA is the same as in the ABTS test.

The main solvent in FRAP experiment is water at pH 3.6. Under these conditions, dihydroacridines are neutral and acridines are protonated. Accordingly, the AOA of acridines was evaluated for the protonated form.

The suggested two-stage antioxidant scheme of dihydroacridines and acridines in the FRAP test is presented in Scheme S2.

As in the ABTS test, two paths are possible: A and B. In path A, the dihydroacridine molecule sequentially donates an electron and an H-atom to two radicals. The first (A1) and second (A2) stages of this path are characterized by the ionization potential IP_A1_ and the bond dissociation enthalpy BDE_A2_, respectively. In path B, the dihydroacridine molecule, on the contrary, first donates an H atom, and then an electron, to two radicals. The first (B1) and the second (B2) stages of this path are characterized by the bond dissociation enthalpy BDE_B1_ and the ionization potential IP_B2_, respectively. IP_A1_, BDE_B1_, and BDE_A2_ were calculated according to Eqs.(S2) through (S4), respectively. IP_B2_ was calculated by Eq. (S6):

| IP_B2_ = *H*(AH_N_^•+^) + *H*(e^-^) – *H*(AH_N_^•^). | (S6) |
| --- | --- |

Whichever way is realized, the enthalpy characteristic of the second stage is low, which means that the first radical reduction stage is immediately followed by the second radical reduction stage.

After the reduction of two radicals, the dihydroacridine molecule transforms into the protonated form of the corresponding acridine, which is the state of acridine at pH 3.6 (see red circle in Scheme S2). As an electron donation by a positively charged protonated acridine is unlikely, only the BDE_p_ values of acridines in their protonated form were calculated for estimation of their AOA values. The BDE_p_ values were calculated for water by Eq. (S7):

| BDE_p_ = *H*(A^*+^) + *H*(H^•^) – *H*(AH_N_^+^) | (S7) |
| --- | --- |

The BDE_p_ values in water are high, which explains the poor AOA of acridines in the FRAP test. All calculated QM characteristics of dihydroacridines and acridines in the FRAP test are presented in Table S4.

## Steric effects

In contrast to the ABTS test, where all dihydroacridines have close experimental AOA values, in the FRAP test, compound **1a** has a slightly higher AOA than compounds **1c** and **1e**. We attribute this to steric effects.

The ABTS molecule is a quasi-one-dimensional object (see Fig.S1a), where the access to the radical does not strongly depend on the steric structure of the antioxidant molecule. The situation is different in the case of [Fe(TPTZ)_2_]^3+^. The cation [Fe(TPTZ)_2_]^3+^ has the shape of a propeller with four blades and a Fe^3+^ atom in the center (Fig.S1b). Such a geometric structure of [Fe(TPTZ)_2_]^3+^ makes it difficult for bulky antioxidant molecules to access it. Compound **1a** with the dimethoxy substituents at phosphorus is a relatively compact structure that fits better between the “blades” of the [Fe(TPTZ)_2_]^3+^complex than compounds **1c** and **1d** with bulky phosphorus substituents.

We surmise that different access of the antioxidant molecules to the radical in the ABTS and FRAP tests explains the observation that compound **1a** has a noticeably higher AOA than compounds **1c** and **1d** in the FRAP test, whereas their AOA values in the ABTS test are almost the same.

# Supplementary Figures and Tables

## Supplementary Figures


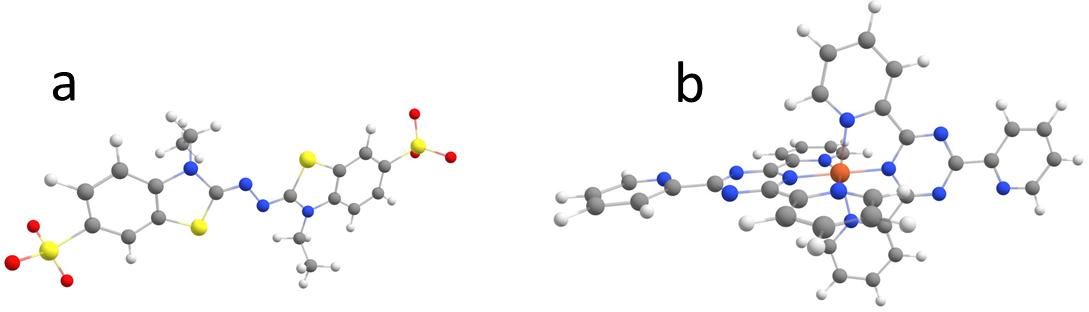


**Supplementary Figure S1.** Optimized geometries of ABTS^•+^ radical-cation (a), and [Fe(TPTZ)_2_]^3+^ cation (b).

## Supplementary Schemes


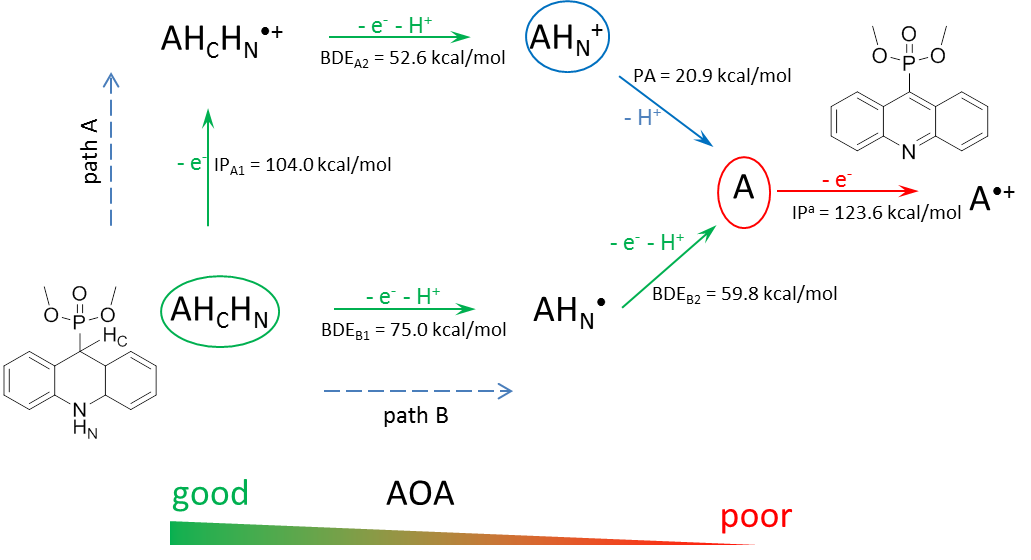


**Supplementary Scheme S1**. Possible antioxidant reactions of dihydroacridines (AH_C_H_N_) and acridines (A) in the ABTS test (ethanol, pH ~4.5). The calculated values of antioxidant characteristics (IP, BDE) are given for compounds **1a** and **2a**. The good antioxidants dihydroacridines (AH_C_H_N_) are circled in green, the poor antioxidants acridines (A) are circled in red, and the protonated form of acridines is circled in blue.


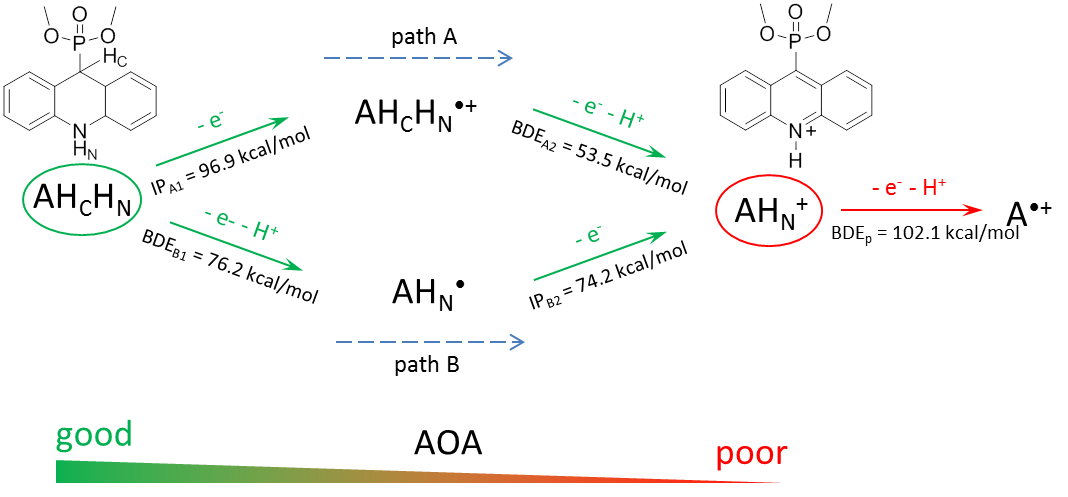


**Supplementary Scheme S2.** Possible antioxidant reactions of dihydroacridines and acridines in the FRAP test (water, pH 3.6). The calculated values of antioxidant characteristics (IP, BDE) are given for compounds **1a** and **2a**. The good antioxidants dihydroacridines (AH_C_H_N_) are circled in green, the poor antioxidants acridines in the protonated form (AH_N_^+^) are circled in red.

## Supplementary Tables

**Supplementary** **Table S1.** Estimated binding affinities of compounds **1d,e** and **2d,e** to Aβ_42_ conformers (PDB ID 1IYT) and their differences.

| Cmpd | PDB 1IYT conformer | | | | | | | | | |
| --- | --- | --- | --- | --- | --- | --- | --- | --- | --- | --- |
|  | 1 | 2 | 3 | 4 | 5 | 6 | 7 | 8 | 9 | 10 |
| **1d** | -5.6 | -5.95 | -5.34 | -5.26 | -5.58 | -5.2 | -5.11 | -5.88 | -5.68 | -5.33 |
| **1e** | -6.18 | -6.37 | -6.07 | -6.14 | -6.05 | -5.54 | -5.92 | -6.58 | -6.95 | -6.56 |
| **2d** | -4.86 | -4.85 | -5.04 | -4.79 | -5.63 | -4.99 | -5.03 | -5.3 | -5.4 | -4.91 |
| **2e** | -5.45 | -5.72 | -5.53 | -5.29 | -5.71 | -5.64 | -5.36 | -6.13 | -6 | -5.67 |
| ΔG_AD,bind_(**1**) - ΔG_AD,bind_(**2**), kcal/mol | | | | | | | | | | |
| **1d**/**2d** | -0.74 | -1.1 | -0.3 | -0.47 | **0.05** | -0.21 | -0.08 | -0.58 | -0.28 | -0.42 |
| **1e**/**2e** | -0.73 | -0.65 | -0.54 | -0.85 | -0.34 | **0.1** | -0.56 | -0.45 | -0.95 | -0.89 |

**Supplementary Table S2.** Number of contacts (4 Å cutoff) between atoms of compounds **1d,e** and **2d,e** and atoms of Aβ_42_ conformers (PDB ID 1IYT) in complexes obtained by molecular docking, and their differences.

| Cmpd | PDB 1IYT conformer | | | | | | | | | |
| --- | --- | --- | --- | --- | --- | --- | --- | --- | --- | --- |
|  | 1 | 2 | 3 | 4 | 5 | 6 | 7 | 8 | 9 | 10 |
| **1d** | 201 | 196 | 193 | 178 | 230 | 186 | 191 | 198 | 209 | 187 |
| **1e** | 205 | 218 | 212 | 164 | 204 | 176 | 205 | 228 | 232 | 186 |
| **2d** | 197 | 196 | 182 | 163 | 166 | 166 | 148 | 178 | 222 | 166 |
| **2e** | 119 | 145 | 165 | 111 | 155 | 199 | 212 | 170 | 158 | 144 |
| N_cont,βA-ligand_(**1**) - N_cont,βA-ligand_(**2**) | | | | | | | | | | |
| **1d**/**2d** | 4 | 0 | 11 | 15 | 64 | 20 | 43 | 20 | -13 | 21 |
| **1e**/**2e** | 86 | 73 | 47 | 53 | 49 | -23 | -7 | 58 | 74 | 42 |

**Supplementary Table S3.** Experimental AOA of dihydroacridines and acridines in the ABTS test and their calculated antioxidant characteristics.

| compound | ABTS test | calculations in ethanol solvent | | | |  |
| --- | --- | --- | --- | --- | --- | --- |
|  | TEAC | IP_A1_, kcal/mol | BDE_B1_, kcal/mol | BDE_A2_, kcal/mol | BDE_B2_, kcal/mol | |
|   **1a** | 0.99 ± 0.04 | 104.0 | 75.0 | 52.6 | 59.8 | |
|   **1c** | 1.13 ± 0.05 | 104.6 | 75.1 | 52.3 | 60.3 | |
|   **1e** | 0.9 ± 0.03 | 103.7 | 79.2 | 52.7 | 58.7 | |
|   **2a** | n.a. | 123.6 | n.av. | n.av. | n.av. | |
|   **2c** | n.a. | 124.5 | n.av. | n.av. | n.av. | |
|   **2e** | n.a. | 121.7 | n.av. | n.av. | n.av. | |
| n.a. – not active ; n.av. – not available; n.c. – not calculated | | | | | | |

**Supplementary Table S4.** FRAP AOA of dihydroacridines and acridines and their calculated antioxidant characteristics.

| compound | TE | IP_A1_, kcal/mol | BDE_B1_, kcal/mol | BDE_A2_, kcal/mol | IP_B2_, kcal/mol | BDE_p_, kcal/mol |
| --- | --- | --- | --- | --- | --- | --- |
|   **1a** | 1.60 ± 0.01 | 96.9 | 76.2 | 53.5 | 74.2 | n.av. |
|   **1c** | 1.13 ± 0.02 | 97.1 | 76.2 | 53.7 | 76.6 | n.av. |
|   **1e** | 1.10 ± 0.01 | 96.0 | 79.2 | 54.0 | 70.9 | n.av. |
|   **2a** | 0.08 ± 0.02 | n.c. |  | n.av. |  | 102.1 |
|   **2c** | n.a. | n.c. |  | n.av. |  | 103.2 |
|   **2e** | n.a. | n.c. |  | n.av. |  | 103.4 |
| n.a. – not active ; n.av. – not available; n.c. – not calculated | | | | | | |

# References

Gowland, J.A., and Schmid, G.H. (1969). Two linear correlations of pKavs. solvent composition. *Can. J. Chem.* 47**,** 2953-2958. DOI:10.1139/v69-493.

Marković, Z., Tošović, J., Milenković, D., and Marković, S. (2016). Revisiting the solvation enthalpies and free energies of the proton and electron in various solvents. *Comput. Theor. Chem.* 1077**,** 11-17. DOI:10.1016/j.comptc.2015.09.007.

Pan, X., Wang, H., Li, C., Zhang, J.Z.H., and Ji, C. (2021). MolGpka: A Web Server for Small Molecule pKa Prediction Using a Graph-Convolutional Neural Network. *J. Chem. Inf. Model.* 61**,** 3159-3165. DOI:10.1021/acs.jcim.1c00075.

Park, C., Atalla, V., Smith, S., and Yoon, M. (2017). Understanding the Charge Transfer at the Interface of Electron Donors and Acceptors: TTF-TCNQ as an Example. *ACS Appl. Mater. Interfaces* 9**,** 27266-27272. DOI:10.1021/acsami.7b04148.
